# Stenting versus gastrojejunostomy for management of malignant gastric outlet obstruction: comparison of clinical outcomes and costs

**DOI:** 10.1007/s00464-012-2301-9

**Published:** 2012-05-02

**Authors:** Ann Roy, Micheline Kim, John Christein, Shyam Varadarajulu

**Affiliations:** 1 Health Economics and Reimbursement, Boston Scientific Corporation, Natick, MA USA; 2 Department of Surgery, University of Alabama at Birmingham, Birmingham, AL USA; 3 Department of Medicine, Gastroenterology and Hepatology, University of Alabama at Birmingham, Birmingham, AL USA; 4 Health Economics and Reimbursement, Boston Scientific Corporation, 100 Boston Scientific Way, M11, Marlborough, MA 01752 USA

**Keywords:** Duodenal stenting, Gastrojejunostomy, Costs, Health resource use

## Abstract

**Background:**

Although endoscopic stenting is increasingly performed, surgical gastrojejunostomy (GJ) is still considered the gold standard for relief of malignant gastric outlet obstruction (GOO). The aim of this study is to compare clinical outcomes and hospital costs between patients undergoing GJ or stenting for management of malignant GOO.

**Methods:**

A retrospective claims analysis of the Medicare (MedPAR) database was conducted to identify all inpatient hospitalizations for GJ or endoscopic stenting for malignant GOO during 2007–2008. The main outcome measure evaluated using the MedPAR database was a comparison of the total length of hospital stay (LOS) and costs associated with both techniques. As MedPAR is a claims database that does not provide outcomes at patient level, a single-institution retrospective study was conducted to compare the rates of technical and treatment success, post-procedure LOS, and delayed complications per patient between the two techniques.

**Results:**

The MedPAR claims data evaluated 425 stenting and 339 GJ hospitalizations. Compared with GJ, median LOS (8 vs. 16 days; *p* < 0.0001) and median cost (US $15,366 vs. US $27,391; *p* < 0.0001) per claim were both significantly lower for stenting. Stenting was more commonly performed at urban versus rural hospitals (89 % vs. 11 %; *p* < 0.0001), teaching versus non-teaching hospitals (59 % vs. 41 %, *p* = 0.0005), and academic institutions (56 % vs. 44 %; *p* = 0.0157). The institutional patient data analysis included 29 patients who underwent stenting and 75 who underwent surgical GJ. While both modalities were technically successful and relieved gastric outlet obstruction in all cases, compared with surgical GJ, the median post-procedure LOS was significantly lower for enteral stenting (1.5 vs. 10.7 days, *p* < 0.0001). There was no difference in rates of delayed complications between stenting and surgical GJ (13.8 % vs. 6.7 %; *p* = 0.26).

**Conclusions:**

While the technical and clinical outcomes of surgical GJ and endoscopic stenting appear comparable, stent placement is less costly and is associated with shorter length of hospital stay. Dissemination of endoscopic stenting beyond teaching, academic hospitals located in urban areas as a treatment for malignant GOO is important given its implications for patient care and resource utilization.

Gastric outlet obstruction (GOO) is a common symptom, occurring in 15–20 % of patients with locally advanced gastrointestinal cancer [1–3]. Clinical manifestations of GOO include nausea, vomiting, and dehydration. Traditionally, surgical gastrojejunostomy (GJ) has been the standard treatment approach for these patients. Although GJ relieves symptoms in almost all patients, the procedure is associated with morbidity of 10–16 % and mortality of up to 7 % [4–6]. Also, post-operatively, most patients suffer delayed gastric emptying that is often associated with prolonged hospital stay [7]. Although laparoscopic GJ has been introduced as a less invasive alternative to open GJ, the technique still carries substantial risk and is not widely available [8–10].

Numerous studies have shown that self-expandable metal stent (SEMS) placement is a relatively simple and safe alternative to surgical GJ for management of malignant GOO [10–12]. The procedure is associated with a technical success rate of greater than 95 %, and a majority of patients can tolerate oral intake following stent deployment [11]. Also, compared with surgery, patients undergoing SEMS placement have shorter length of hospital stay [4, 12]. A disadvantage of SEMS is the high rate of late complications caused by stent migration and occlusion [2]. Also, SEMS are expensive and it is unclear whether their use is less costly when compared with surgical GJ. Although direct cost studies have shown that SEMS placement is less costly than surgery, the general applicability of the data is debatable given the small number of patients enrolled in each of these single-institution trials [13–15].

The objectives of the present study are to (1) compare the hospital costs and length of stay (LOS) at a national level by using a claims database and (2) compare the clinical outcomes at a patient level by conducting a single-institution retrospective study for patients undergoing surgical GJ versus stenting for management of malignant GOO.

## Patients and methods

This study was conducted in two parts: First, Medicare Provider Analysis and Review (MedPAR) inpatient hospitalization data were utilized to evaluate hospital costs and length of stay (LOS) for patients who underwent gastrojejunostomy or endoscopic duodenal stenting for relief of malignant gastric outlet obstruction. Second, since clinical outcomes may not be evaluated using MedPAR, a retrospective study was conducted to evaluate patient outcomes at an institutional level.

### MedPAR data source

A retrospective analysis was conducted utilizing 2007–2008 MedPAR data. This database contains complete inpatient hospitalization claim records for the entire US Medicare population of 44 million (2007)–45.5 million (2008) covered lives. MedPAR claims contain information on patient demographics including age, sex, diagnosis, and comorbidities. In addition, information regarding hospitalization such as LOS, diagnostic testing, therapeutic procedures, and hospital charges is provided. The MedPAR dataset was linked to the provider of services (POS) file, which provides the geographic and demographic information for the hospitals where the claims were generated (location, associations, teaching status, and number of beds).

#### Patient population

The study population consisted of all unique hospitalization claims for a GJ procedure or endoscopic placement of a duodenal stent. Claims were included based on primary diagnosis and procedure. GJ claims were identified by an obstruction of duodenum diagnosis defined as ICD-9-CM diagnosis code 537.3 reported with any one of the following cancer diagnoses: pancreas, gallbladder, bile ducts, biliary tract, small intestine, or duodenum. In addition, the claim had to have any one of the following surgical GJ ICD-9-CM procedure codes reported: 44.38 or 44.39.

In 2007–2008, there was no unique ICD-9-CM procedure code to report for endoscopic placement of a duodenal stent. As such, the following proxy was developed to identify stent placement claims: the claim had to have an obstruction of duodenum with cancer diagnosis reported, as described above. In addition, the stenting claim had to have the following endoscopy of small intestine ICD-9-CM procedure code 45.13 reported with revenue code “0278: Medical/Surgical Supplies other implant.” Claims where both GJ and stent placement procedures were reported together were excluded from the analysis.

#### Outcome measures

Using the MedPAR claims data, the total LOS from admission to discharge and the total costs associated with each technique were compared. As per the Medicare cost report, the hospitalization costs were derived by applying the appropriate cost-to-charge ratio to the charges reported in MedPAR. A secondary analysis was also conducted to analyze demographics of the hospitals in which the procedures were performed.

### Institutional data

As MedPAR is a claims database that does not provide outcomes at a patient level, a single-institution retrospective case-control study was conducted where each patient who underwent stent placement was matched with two gastrojejunostomy patients having procedures in the period 2006–2008.

#### Patient population

A retrospective analysis was conducted of consecutive patients (>19 years of age) who underwent surgical GJ or duodenal stenting for management of malignant GOO. Patients were identified from the endoscopy and surgery databases. Inclusion criteria were: patient age >19 years, underlying diagnosis of cancer of the pancreaticobiliary system, and procedures undertaken for relief of GOO. Excluded were patients who underwent GJ or stenting for benign diseases. The medical records of all study subjects were reviewed for patient demographics, clinical presentation, comorbidities, laboratory investigations, cancer staging, and radiological investigations.

#### Duodenal stenting

All SEMS (WALLSTENT or WALLFLEX; Boston Scientific Corp., Natick, MA) were deployed under fluoroscopic guidance with the patient in left lateral position using a combination of intravenous midazolam and meperidine. The stents measured 22 mm in the body and 27 mm in the proximal flare, and were 6, 9, or 12 cm in length. At gastroscopy, a 0.035 in guide wire was first advanced across the stricture. A 5Fr endoscopic retrograde cholangiopancreatoscopy (ERCP) cannula was then advanced over the guide wire, and contrast was injected to assess the length of the stricture. The SEMS delivery system was then advanced over the guide wire, and after satisfactory positioning of the delivery catheter was confirmed by fluoroscopy, the SEMS was deployed.

#### Surgical GJ

Briefly, after making a midline incision, a small opening was made into both the posterior wall of the stomach and the jejunal loop with a Harmonic scalpel. The jaws of an Endo-GIA stapler (3.5 mm/60 mm; US Surgical, Norwalk, CT) were inserted into the enterotomies, and a wide gastrojejunostomy was created by three firings of the stapler**.** The staple line was then carefully inspected for bleeding, and the enterotomies were closed with a running suture.

#### Outcome measures

The rates of treatment success, complications, re-interventions, and length of post-procedure hospital stay were compared between each treatment modality.

#### Consent

All patients provided informed consent to undergo the procedures, and the study was approved by the University of Alabama at Birmingham Institutional Review Board.

### Statistical analysis

#### MedPAR data

All analyses were performed using SAS version 9.2 (SAS Institute, Cary, NC). Discrete data are reported as frequencies, and continuous data are reported as median/mean. Using a chi-square test, the patient demographics and comorbidities reported on the claims were compared across the GJ and stenting cohorts. Covariates were not adjusted for in the analysis of health resources due to limitations associated with using MedPAR data.

#### Institutional data

Statistical analysis was performed by using Stata 9.2 (StataCorp LP, College Station, TX). Patient demographics and disease characteristics were compared across the two groups (SEMS stenting and GJ). A two-sample *t* test was used to compare the means of the continuous variables such as age, albumin levels, and Charlson score. A chi-square test was used to compare the proportions of disease characteristics (Table 1) and outcome measures (Table 2) across the two groups.

**Table 1:** Patient characteristics for stent and surgery groups (institutional data)

	Stent (*n* = 29)	Surgery (*n* = 75)	*p* value
Age (mean years)	59.6	62.9	0.2026
Sex (% females)	48.3	58.7	0.3387
Race (% Black)	34.5	27.03	0.4538
Metastasis (% yes)	68.9	80.0	0.3305
Charlson score (mean)	5.97	4.84	0.0305*

* *p* < .05

**Table 2:** Clinical outcomes for stent and surgery groups (institutional data)

	Stent (*n* = 29)	Surgery (*n* = 75)	*p* value
Relief of obstruction (%)	100	100	1
Intra-procedural complications (*n*, %)	0 (0)	0 (0)	1
Delayed complications (*n*, %)	4 (13.8)	5 (6.7)	0.26
Post-procedure hospital stay (mean days)	1.52	10.72	<0.0001*

* *p* < .05

## Results

### MedPAR data

A total of 339 GJ and 425 duodenal stent placement claims met the study inclusion criteria. The age, gender, and comorbidities of patients in both cohorts are presented in Table 3. There was no significant difference in gender distribution between the two groups. With the exception of cerebrovascular disease, diabetes without complications, and moderate/severe liver disease, there was no significant difference between the two cohorts for comorbid conditions. Each of these comorbidities was more prevalent in the stenting group.

**Table 3:** Baseline demographics and comorbid conditions of the stent placement and gastrojejunostomy patient populations as reported in 2007–2008 MedPAR claims (MedPAR data)

	Stenting claims (*n* = 425) %	Gastrojejunostomy claims (*n* = 339) %	*p* value
Age group (years)			
45–64	8	6	0.176
65–69	16	22	0.019
70–74	17	20	0.301
75–79	20	19	0.983
80–84	18	20	0.564
85–89	13	9	0.099
>89	7	3	0.011
Female	54	50	0.275
Comorbid conditions			
Congestive heart failure	8	9	0.494
Chronic obstructive pulmonary disease	11	7	0.067
Cerebrovascular disease	3	1	0.055
Diabetes without complications	21	14	0.007
Diabetes with complications	2	1	0.119
Myocardial infarction	3	2	0.393
Moderate/severe liver disease	3	0	0.007
Peripheral vascular disease	3	1	0.115
Renal disease	4	4	0.818

**Fig. 1:**
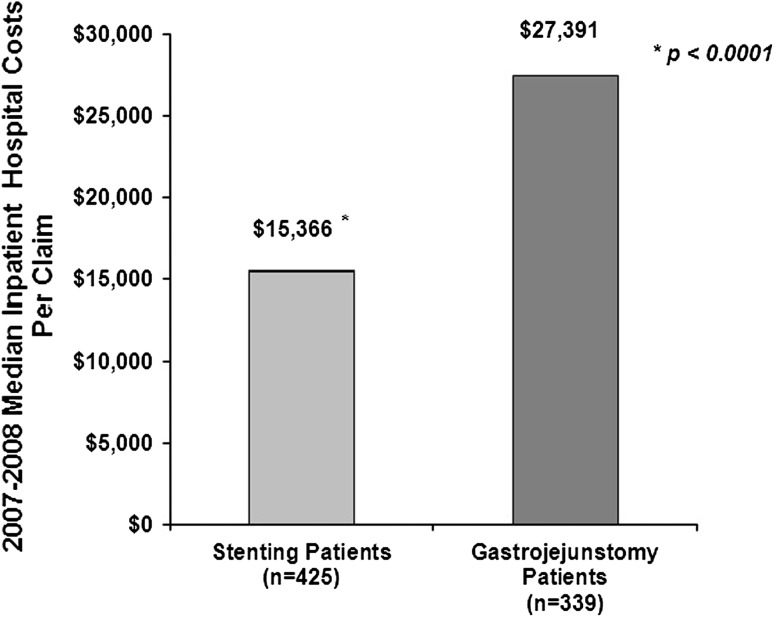
Median inpatient hospital costs per claim for duodenal stenting claims versus gastrojejunostomy claims (MedPAR data)

Median aggregate hospital days or total LOS from admission to discharge was 8 versus 16 days for duodenal stent placement and GJ claims, respectively (*p* < 0.0001). Stent placement claims had significantly lower total median hospital costs per claim than GJ claims (US $15,366 vs. US $27,391; *p* < 0.0001) (Fig. 1). Mean hospital costs for the stent placement cohort were US $20,133 versus US $35,444 for the GJ cohort (*p* < 0.0001).

Stent placement was more commonly performed in urban versus rural hospitals (89 % vs. 11 %; *p* < 0.0001), teaching versus non-teaching hospitals (59 % vs. 41 %; *p* = 0.0005), and academic institutions (56 % vs. 44 %; *p* = 0.0157).

### Institutional data

The single-institution retrospective study involved 29 patients who underwent SEMS placement and 75 patients who underwent surgical GJ for malignant GOO. Table 1 displays demographics and disease characteristics of all patients at presentation. There was no significant difference between cohorts with regard to patient demographics or presence of metastasis. Patients who underwent stent placement had significantly higher comorbidities as indicated by their Charlson scores.

Surgical GJ and SEMS placements were successful in relieving the obstruction in all patients. While the mean duration of post-procedure LOS was significantly shorter (1.52 vs. 10.72 days, *p* < 0.0001), there was no difference in pre-procedure LOS (2 vs. 3.4 days, *p* = 0.52) between patients who underwent SEMS placements versus surgery, respectively. Although there were no complications in the immediate postintervention period among patients who underwent SEMS placement, six patients in the surgical cohort encountered complications (0 % vs. 8 %, *p* = 0.18) that included death from sepsis (*n* = 1, post-operative day 9), wound infection (*n* = 2), and delayed gastric emptying that required placement of a temporary nasoenteric feeding tube via the surgical anastomosis (*n* = 3). Delayed complications were encountered in four patients in the SEMS cohort [median (range): 97 (83–139) days] compared with five patients in the surgical cohort [median (range): 41 (26–61) days] (13.8 vs. 6.7 %; *p* = 0.26). Complications in the SEMS cohort included stent occlusion in three patients and small bowel perforation from a migrated stent in one. Two of three patients with stent occlusion were managed by placement of additional stents, and one patient was treated with balloon dilation; the small bowel perforation was managed by surgical removal of the stent with repair of the perforation and a surgical GJ. Three of four patients admitted for delayed complications were discharged within 24 hours of reintervention, and the patient who underwent surgery required a 7 day hospitalization. The mean duration of hospitalization for delayed complications was 2.5 days (range 1–7 days) for the stenting cohort. Delayed complications in the surgical cohort included bowel obstruction that required exploratory laparotomy with lysis of adhesions in three patients and delayed gastric emptying due to an anastomotic stricture that required gastroscopy with dilation in two patients. The mean duration of hospitalization for delayed complications was 5.2 days (range 2–9 days) for the surgical cohort.

Of the 29 patients who underwent SEMS placement, at median follow-up of 5 months (range 1–11 months), 25 patients had died and 4 were lost to follow-up. Of the 75 patients who underwent surgical GJ, at median follow-up of 6 months (range 1–13 months), 67 had died, 3 were receiving palliative chemotherapy, and 5 were lost to follow-up. There was no difference in median duration of survival between the SEMS and surgical GJ cohorts (118 vs. 132 days, respectively; *p* = 0.67).

## Discussion

The main objective of a palliative procedure in patients with malignant GOO is to restore their ability to eat. Several studies have shown that SEMS placement is a safe and effective alternative to surgery [10–12]. A comprehensive review of 32 case series including 606 patients unable to take oral intake reported successful stent deployment in 97 % of patients, and oral intake was possible in all successful cases, with 87 % of patients capable of eating at least a mechanical soft diet [11]. There are limited reports comparing stenting of the gastric outlet or small intestine with surgical bypass. A small randomized prospective study of 18 patients comparing SEMS placement versus surgical bypass found no difference in survival, complication rates, or gastric emptying at 3 months, but the SEMS group had more rapid restoration of oral intake and shorter mean hospitalization [12]. Similarly, a retrospective comparison of a cohort of 27 patients with pancreatic cancer causing duodenal obstruction treated with endoscopic stenting versus surgical bypass found no difference in survival but median hospital stay of 4 days in the stent group versus 14 days in the surgical group [4]. A prospective nonrandomized study of 36 patients found no difference in overall survival or ability to tolerate food 1 month after stent placement or surgical bypass [13]. In the present study that evaluated claims data of 764 patients, the median LOS per claim was 8 versus 16 days for the SEMS and surgical GJ cohorts, respectively. While total length of hospital stay can be estimated using the MedPAR database, it is not possible to assess the post-procedure LOS. When these clinical outcomes were evaluated at institutional level, the difference in post-procedure LOS was even more pronounced: 1.5 versus 10.7 days. Even in the absence of complications, surgical patients required lengthier post-procedure hospitalization for pain management and tolerance of oral intake. Similar to prior reports [4, 10–12], our analysis of institutional data found no significant difference in rates of treatment success, procedural or delayed complications, or median duration of survival between the two groups.

While three prior single-institution studies, two from Europe and one from the USA, have shown that stent placement was significantly less costly than surgery, this has never been examined from a national perspective. In the present study, the median cost per claim was US $12,025 lower for patients undergoing SEMS placement. These findings, involving 425 SEMS and 339 surgical claims, are more realistic representations of the costs associated with both treatment modalities in the US population.

Analysis of the claims data revealed that, despite its inherent clinical advantages and cost savings, duodenal stenting is more commonly performed at large, urban, teaching hospitals. We speculate that, in large teaching institutions, better collaboration between the disciplines of medical gastroenterology and general surgery results in more patients undergoing SEMS placement. In smaller and rural hospitals, patients are probably managed based on the manner in which they are triaged at admission: those admitted to the gastroenterology service are more likely to undergo stent placement, whereas those admitted to the surgical service undergo resection. It is our opinion that, at least for patients with multiple comorbidities and poor functional status, duodenal stent placement should be the favored initial treatment approach, as these patients are poor operative candidates. More education and training is needed to propagate appropriate use of stenting in patients presenting with malignant GOO.

Our study suffers from some limitations. As it would have been ideal to have conducted a longitudinal study evaluating the two cohorts from the initial procedure to death, tracking procedures performed and associated costs, the inability to conduct a longitudinal analysis is one of the limitations of analyses of the MedPAR claims database. There are no unique patient identifiers to track the same patient over time. The MedPAR database only allows for cross-sectional studies to be performed. In addition, outcomes data, such as procedural success and complication rates, cannot be evaluated using a claims database. It is also possible that some of these patients who underwent surgical GJ had failed attempts at prior stent placement. At an institutional level, as patients were not followed prospectively, it is likely that some minor adverse events were not captured. Also, it is possible that outcomes were influenced by patient characteristics in each group. However, this may not be a major limitation as patients undergoing SEMS had higher Charlson scores (5.97 vs. 4.84, *p* = 0.03). In addition, this retrospective study examined a small sample of patients from a single institution. As a result, the ability to generalize these findings to a national sample is limited and is not the intent of this study. Being a retrospective study, we could not present data on the rates of technical failure in the SEMS cohort or on the number of patients who failed stent placement and subsequently underwent surgery.

In conclusion, while the technical and clinical outcomes of GJ and stent placement appear comparable in relieving obstruction, stent placement is less costly and is associated with shorter LOS. Dissemination of stent placement beyond teaching hospitals located in urban areas as a treatment for malignant GOO is important given its implications for patient care and resource use.
